# The safety and clinical outcomes of endovascular treatment versus microsurgical clipping of ruptured anterior communicating artery aneurysms: a 2-year follow-up, multicenter, observational study

**DOI:** 10.3389/fneur.2024.1389950

**Published:** 2024-05-23

**Authors:** Minghao Yang, Yang Li, Jia Li, Xiuhu An, Hongwen Li, Bangyue Wang, Yan Zhao, Xiaowei Zhu, Changkai Hou, Linchun Huan, Xinyu Yang, Jianjun Yu

**Affiliations:** ^1^Department of Neurosurgery, Tianjin Medical University General Hospital, Tianjin, China; ^2^Department of Cerebrovascular Disease, The Second Affiliated Hospital of Guilin Medical University, Guangxi Zhuang, China; ^3^Department of Neurosurgery, Baoding No.1 Central Hospital, Hebei, China; ^4^Department of Neurosurgery, Linyi People’s Hospital, Shandong, China

**Keywords:** AComA aneurysm, endovascular treatment, microsurgical clipping, propensity score matching, vascular disorders

## Abstract

**Background and objective:**

Current data on the optimal treatment modality for ruptured anterior communicating artery (AComA) aneurysms are limited. We conducted this multicenter retrospective study to evaluate the safety and clinical outcomes of endovascular treatment (EVT) and microsurgical clipping (MC) for the treatment of ruptured AComA patients.

**Methods:**

Patients with ruptured AComA aneurysms were screened from the Chinese Multicenter Cerebral Aneurysm Database. Propensity score matching (PSM) was used to adjust for baseline characteristic imbalances between the EVT and MC groups. The safety outcomes included total procedural complications, procedure-related morbidity/death and remedial procedure for complication. The primary clinical outcome was 2-year functional independence measured by the modified Rankin scale (mRS) score.

**Results:**

The analysis included 893 patients with ruptured AComA aneurysms (EVT: 549; MC: 346). PSM yielded 275 pairs of patients in the EVT and MC cohorts for comparison. Decompressive craniectomy being more prevalent in the MC group (19.3% vs. 1.5%, *p <* 0.001). Safety data revealed a lower rate of total procedural complications (odds ratio [OR] = 0.62, 95% CI 0.39–0.99; *p =* 0.044) in the EVT group and similar rates of procedure-related morbidity/death (OR = 0.91, 95% CI 0.48–1.73; *p =* 0.880) and remedial procedure for complication (OR = 1.35, 95% CI 0.51–3.69, *p =* 0.657) between the groups. Compared with that of MC patients, EVT patients had a greater likelihood of functional independence (mRS score 0–2) at discharge (OR = 1.68, 95% CI 1.14–2.50; *p =* 0.008) and at 2 years (OR = 1.89, 95% CI 1.20–3.00; *p =* 0.005), a lower incidence of 2-year all-cause mortality (OR = 0.54, 95% CI 0.31–0.93; *p =* 0.023) and a similar rate of retreatment (OR = 1.00, 95% CI 0.23–4.40; *p =* 1.000).

**Conclusion:**

Clinical outcomes after treatment for ruptured AComA aneurysms appear to be superior to those after treatment with MC, with fewer overall procedure-related complications and no increase in the retreatment rate. Additional studies in other countries are needed to verify these findings.

## Introduction

The anterior communicating artery (AComA) is one of the most common sites for intracranial aneurysms, and approximately 40% of aneurysmal subarachnoid hemorrhages (SAH) in adults are caused by aneurysm rupture at this location (1, 2). Both microsurgical clipping (MC) and endovascular treatment (EVT) are effective approaches for treating AComA aneurysms. However, due to the deep and midline position of the AComA, frequent anatomical variations, the presence of critical structures, and intricate vascular morphology, both MC and EVT continue to present unique technical challenges.

Traditionally, clinicians choose the treatment modality based on anatomical criteria and their own expertise. Wide-necked aneurysms, tiny aneurysms, and aneurysms associated with compressive hematomas are often assigned to surgical clipping. In recent years, with the ongoing progress in interventional techniques and materials, anatomical factors have become less restrictive for EVT (3). EVT has been increasingly considered a preferred option for ruptured AComA aneurysms (4). However, the existing evidence is insufficient to demonstrate the clear superiority of EVT in terms of safety and efficacy. Early clinical studies revealed no significant differences in clinical outcomes or safety between EVT and MC for ruptured AComA aneurysms (5). In a recent meta-analysis including both ruptured and unruptured aneurysms comprising 18 studies, Sattari et al. reported no differences in clinical outcomes (OR = 0.77, 95% CI 0.49–1.20, *p =* 0.25) or mortality rates (OR = 0.92, 95% CI 0.62–1.36, *p =* 0.66) between the MC and EVT groups, while angiographic outcomes tended to favor MC (retreatment OR = 0.31, 95% CI 0.11–0.89, *p =* 0.03); recurrence (OR = 0.16, 95% CI 0.03, 0.90, *p =* 0.04) (6). Due to the limitations inherent in the original studies, the meta-analysis could not draw definitive conclusions. The optimal treatment modality for this lesion remains uncertain. Therefore, we conducted this multicenter study with the aim of characterizing the safety and clinical outcomes of EVT and MC for ruptured AComA aneurysms, focusing particularly on 2-year clinical outcomes, to provide evidence for treatment decision-making in such patients.

## Methods

### Study design and population

We conducted a retrospective propensity score-matched cohort study using the Chinese Multicenter Cerebral Aneurysm Database (CMAD) (Clinical Trial Registry No. ChiCTR2100054014), a prospective registry in 32 tertiary medical centers across four northern provinces in China. Twelve of these centers enrolled consecutive patients with intracranial aneurysms from 2017 to 2020. We identified all patients diagnosed with ruptured AComA aneurysms from the CMAD with the following inclusion criteria: (1) age > 18 years, (2) AComA aneurysm considered responsible for SAH or intracranial hematoma (ICH), and (3) treated with either EVT or MC. The exclusion criteria were as follows: (1) non-saccular aneurysm; (2) concomitant aneurysm at another location requiring simultaneous or staged treatment, (3) comorbid conditions such as arteriovenous malformations, arteriovenous fistula, and moyamoya disease, (4) treated with a flow-diverter device or endosaccular flow disruption device, (5) dependency before onset (modified Rankin scale [mRS] score > 2), and (6) loss to follow-up.

Approval was obtained from the Ethics Committee of Tianjin Medical University General Hospital (IRB2021-YX-178-01). Considering the observational design of the study, the ethics committee waived the requirement for informed consent. The research strictly adhered to relevant Chinese laws and research guidelines in accordance with the principles of the Helsinki Declaration. The manuscript was written in accordance with the STROBE (Strengthening the Reporting of Observational Studies in Epidemiology) guidelines (Supplementary Table S1) (7).

### Data collection

The following baseline patient information was collected from the database: age, sex, smoking status, World Federation of Neurological Surgeons (WFNS) grade, Hunt–Hess grade, Fisher grade, associated intraventricular hemorrhage (IVH), and associated ICH. Comorbidities, including cancer, kidney disease, liver disease, diabetes, chronic lung disease, hypertension, coronary heart disease, congestive heart failure, and hypertension, were identified and extracted based on the International Classification of Diseases coding system (ICD-9-CM). The Charlson Comorbidity Index (CCI) was used to assess comorbidities (8). Treatment characteristics included the timing of treatment, treatment type, adjunctive procedures (decompressive craniectomy [DC], external ventricular drain [EVD], ventriculoperitoneal [VP] shunt, tracheostomy) and other treatment (lumbar drainage, lumbar puncture and allogeneic blood transfusion).

### Safety outcomes

The safety outcomes were total procedural complications, procedure-related morbidity/death and remedial procedure for complication. Procedural complications included intraprocedural aneurysm rupture, thrombus formation, device-related events (coil protrusion/migration, arterial dissection), postprocedural intracranial hemorrhage, postprocedural symptomatic infarction, postprocedural seizure, and access site complications (puncture site hematoma, retroperitoneal hematoma, subdural hygroma, wound infection and/or breakdown). Remedial procedure for complication including intra-arterial thrombolysis, mechanical thrombectomy, emergency stent implantation, ICH evacuation, and DC. In addition, other medical complications unrelated to the index procedure, including acute hydrocephalus, intracranial infection, pneumonia, ulcer stress bleeding, deep vein thrombosis (DVT), and urinary tract infection (UTI), were also assessed. All the data were collected and extracted by a team of neurosurgery residents and graduate students and were ultimately reviewed individually by two senior neurosurgeons.

### Follow-up and clinical outcomes

During 2022, follow-up assessments were conducted via standardized telephone interviews with patients or their close relatives by specialized physicians unfamiliar with the treatment received. Loss to follow-up was defined as patients who have not visited outpatient clinics since discharge or have lost contact via phone during the year 2022. The primary outcome was functional independence at 2 years, defined as an mRS score of 0 to 2 or equivalent descriptions. Secondary outcomes included functional independence at discharge, in-hospital mortality, rate of 2-year all-cause mortality, the retreatment rate and the length of hospital stay. Survival time was defined as the interval from symptom onset to death. Retreatment referred to any therapeutic intervention for the recurrence of an aneurysm.

### Statistical analysis

Continuous variables are expressed as the mean ± standard deviation (SD) or median (interquartile range [IQR]), and independent sample *t* tests or Mann–Whitney *U* tests were performed based on the normality test results. Categorical variables are presented as numbers (percentages), and comparisons of these data were made using Fisher’s exact test or Pearson’s chi-square test, as appropriate.

PSM was used to adjust for baseline characteristic imbalances between the groups (9). The propensity score was calculated by logistic regression to estimate the probability that a patient would undergo EVT or MC. According to the prior studies (10, 11), following variables were included in the propensity matching model: sex, age, smoking history, hypertension status, Charlson score, Hunt–Hess grade, WFNS grade, Fisher grade, IVH, ICH, recurrent aneurysm, preprocedural rebleeding, and type of hospital (academic or nonacademic). PSM was carried out using a 1:1 nearest-neighbor method with a caliper width of 0.05 without replacement. Patient baseline characteristics were examined before and after PSM. Odds ratios of safety and clinical outcomes were calculated after matching using Fisher’s exact test.

To examine potential heterogeneity of the primary outcome, subgroup analyses were performed within the matched cohorts using univariate logistic regression models. These subgroups included sex, age (<60 vs. ≥60 years), Charlson score (<1 vs. ≥1), WFNS grade (I–III vs. IV–V), Hunt–Hess grade (I–III vs. IV–V), Fisher grade (1–2 vs. 3–4), and hospital type (academic vs. non-academic). The interactions between treatment and the specific subgroup were evaluated, with *p <* 0.05 considered statistically significant.

Sensitivity analysis was performed on the entire dataset. Variables with a significance level of *p <* 0.1 in the univariate analysis were included in a multivariate logistic regression model for adjustment, including smoking, Fisher grade, type of hospital.

Additionally, because PSM can only address measured confounders, we calculated E-values for primary outcome to evaluate the strength of potential unmeasured confounding on the observed associations (12).

All bilateral tests used a statistical significance threshold of *p =* 0.05. All calculations were performed using R software (version 4.2.3; R Foundation for Statistical Computing, Vienna, Austria) and SPSS version 26 (IBM, Armonk, New York, USA).

## Results

### Patient characteristics and PSM

**Figure 1:**
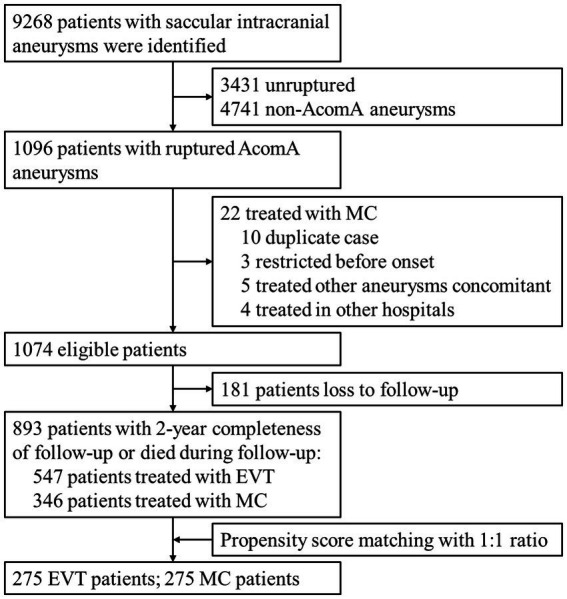
Flow chart of patient selection. AComA, anterior communicating artery; EVT, endovascular treatment; MC, microsurgical clipping.

A total of 1,096 records were initially screened, and after excluding ineligible records, 1,074 patients with rupture AComA aneurysms and treated with aneurysm repair surgeries were identified. Among these patients, 181 were excluded due to loss to follow-up. The distribution of patients lost to follow-up was balanced between the EVT and MC groups, and the baseline characteristics and discharge outcomes were similar to those of the entire population (Supplementary Table S2). Ultimately, a total of 893 patients met the inclusion criteria for the final cohort. Patient selection is detailed in Figure 1.

In the entire cohort, the mean age was 55.3 ± 11.0 years (range, 23–81 years), and 47.4% of the patients were female. Most patients (82.0%) had a Charlson score of 0, and 85.7% of patients were admitted with good-grade SAH (Hunt–Hess grades I–III). Associated IVH was present in 15.9% of patients, and ICH was present in 11.3% of patients. Among them, 547 patients (61.3%) underwent EVT, and 346 (38.7%) underwent MC. Before matching, there were some differences in baseline characteristics between the EVT and MC groups. Specially, a higher proportion of patients in the EVT group were smokers compared to the MC group (24.7% vs. 17.1%, *p =* 0.008). The proportion of patients in the EVT group who were hospitalized in academic hospitals was 22.5%, while it was 51.7% in the MC group (*p <* 0.001). PSM yielded 275 pairs of patients between the EVT and MC groups. After PSM, all baseline characteristics were well distributed between the two groups (Table 1).

**Table 1:** Baseline characteristics before and after propensity score matching.

Characteristic	Before matching			After matching		
	EVT (*n* = 547)	MC (*n* = 346)	*p-*value	EVT (*n* = 275)	MC (*n* = 275)	*p-*value
Sex male	296 (54.1)	174 (50.3)	0.272	141 (51.3)	143 (52.0)	0.932
Age, years	55.7 (10.9)	54.7 (11.2)	0.210	55.5 (10.3)	54.5 (10.8)	0.246
Smoking	135 (24.7)	59 (17.1)	0.008	46 (16.7)	45 (16.4)	1.000
Hypertension	278 (50.8)	188 (54.3)	0.336	146 (53.1)	154 (56.0)	0.549
**Charlson score**						
0	452 (82.6)	280 (80.9)	0.774	222 (80.7)	223 (81.1)	0.774
1–2	89 (16.3)	61 (17.6)		48 (17.5)	49 (17.8)	
≥3	6 (1.1)	5 (1.4)		5 (1.8)	3 (1.1)	
**HH grade**						
I–III	476 (87.0)	289 (83.5)	0.170	237 (86.2)	236 (85.8)	1.000
IV–V	71 (13.0)	57 (16.5)		38 (13.8)	39 (14.2)	
**WFNS grade**						
I–III	420 (76.8)	262 (75.7)	0.747	214 (77.8)	213 (77.5)	1.000
IV–V	127 (23.2)	84 (24.3)		61 (22.2)	62 (22.5)	
**Fisher grade**						
1–2	371 (67.8)	216 (62.4)	0.111	184 (66.9)	175 (63.6)	0.474
3–4	176 (32.2)	130 (37.6)		91 (33.1)	100 (36.4)	
Recurrence	5 (0.9)	4 (1.2)	0.741	3 (1.1)	3 (1.1)	1.000
Preop rebleeding	5 (0.9)	6 (1.7)	0.353	2 (0.7)	6 (2.2)	0.285
IVH	83 (15.2)	59 (17.1)	0.454	40 (14.5)	44 (16.0)	0.722
ICH	58 (10.6)	43 (12.4)	0.448	33 (12.0)	33 (12.0)	1.000
Academic hospital	123 (22.5)	179 (51.7)	<0.001	115 (41.8)	112 (40.7)	0.863

EVT, endovascular treatment; MC, microsurgical clipping; SD, standardized difference; HH, Hunt-Hess scale; WFNS, World Federation of Neurological Surgeons clinical grading scale; IVH, Intraventricular hemorrhage; ICH, intracerebral hemorrhage.

Unless indicated otherwise, data are presented as the number of patients (%).

### Treatment characteristics

Treatment characteristics of the matched cohort are summarized in Table 2. Within 24 h of onset, 136/275 (49.5%) and 98/275 (35.6%) patients in the EVT and MC groups underwent aneurysm repair surgery (*p =* 0.001). Within the EVT cohort, 165/275 (60.0%), 83/275 (30.2%) and 27/275 (9.8%) patients underwent stand-alone coiling, stent-assisted coiling (SAC) and balloon-assisted coiling (BAC), respectively. All patients in the MC cohort underwent surgical clipping. DC (19.3% vs. 1.5%, *p <* 0.001) and tracheostomy (8.7% vs. 2.9%, *p =* 0.005) were more common in the MC group, while EVD (14.9% vs. 12.0%, *p =* 0.382) and VP shunt (4.4% vs. 4.0%, *p =* 1.000) were similar between the two groups. The distribution of reasons for DC in the MC group was summarized in Supplementary Table S3.

**Table 2:** Comparison of treatment characteristics between the EVT and MC groups.

Variable	EVT (*n* = 275)	MC (*n* = 275)	*p-*value
**Timing of treatment**			
<24 h	136 (49.5)	98 (35.6)	0.004
24–72 h	82 (29.8)	98 (35.6)	
>72 h	57 (20.7)	79 (28.7)	
**Therapy modalities**			
Clipping		275 (100.0)	NA
Coiling	275 (100.0)		NA
Stand-alone coiling	165 (60.0)		
SAC	83 (30.2)		
BAC	27 (9.8)		
**Other procedures**			
DC	4 (1.5)	53 (19.3)	<0.001
EVD	33 (12.0)	41 (14.9)	0.382
VP shunt	11 (4.0)	12 (4.4)	1.000
Tracheostomy	8 (2.9)	24 (8.7)	0.005
Lumbar drainage	94 (34.2)	81 (29.5)	0.272
Lumbar puncture	126 (45.8)	118 (42.9)	0.548
Transfusion	1 (0.4)	13 (4.7)	0.002

EVT, endovascular treatment; MC, microsurgical clipping; SAC, stent assisted coiling; BAC, balloon assisted coiling; DC, decompressive craniectomy; EVD, external ventricular drainage; VP shunt, Ventriculoperitoneal shunt.

In addition, there was also a lower likelihood of transfusion in the EVT group than that in the MC group (0.4% vs. 4.7%, *p =* 0.002). The rates of lumbar drainage (34.2% vs. 29.5%, *p =* 0.272) and lumbar puncture (45.8% vs. 42.9, *p =* 0.548) were comparable between the groups (Table 2).

### Safety outcomes

There were 39/275 and 58/275 patients in the EVT and MC groups, respectively, who experienced at least one type of procedural complication, resulting in total procedural complication rates of 14.2 and 21.1% (OR = 0.62, 95% CI 0.39–0.99; *p =* 0.044), respectively; the corresponding rates of procedure-related morbidity/death were 8.4 and 9.1% (OR = 0.91, 95% CI 0.48–1.73; *p =* 0.880), respectively. Remedial procedure for complication occurred in 12/275 (4.4%) EVT patients and 9/275 (3.3%) MC patients (OR = 1.35, 95% CI 0.51–3.69; *p =* 0.657). With regard to type of complications, the EVT and MC groups exhibited comparable rates of hemorrhagic complications (OR = 0.83, 95% CI 0.39–1.75; *p =* 0.727) and ischemic complications (OR = 1.10, 95% CI 0.57–2.16; *p =* 0.875). The prevalent complications in the EVT group were thrombosis (4.7%), postprocedural symptomatic infarction (3.6%), intraprocedural aneurysm rupture (2.9%), and postprocedural intracranial hemorrhage (2.9%), and in the MC group were postprocedural symptomatic infarction (8.4%), postprocedural intracranial hemorrhage (4.4%), postprocedural seizure (4.4%), and access site complications (4.4%) (Table 3).

**Table 3:** Procedural complications and other medical complications.

Variable	EVT (*n* = 275)	MC (*n* = 275)	OR (95% CI)	*p-*value
Total procedural complications	39 (14.2)	58 (21.1)	0.62 (0.39–0.99)	0.044
**Complications by timing**				
Intraprocedural ruptured	8 (2.9)	10 (3.6)	0.79 (0.27–2.27)	0.811
Thrombosis	13 (4.7)			NA
Device-related events	3 (1.1)			NA
Postprocedural intracranial hemorrhage	8 (2.9)	12 (4.4)	0.66 (0.23–1.78)	0.495
New symptom infarction	10 (3.6)	23 (8.4)	0.41 (0.17–0.93)	0.030
Postprocedural seizure	4 (1.5)	12 (4.4)	0.32 (0.08–1.09)	0.073
Access site complication	3 (1.1)	12 (4.4)	0.24 (0.04–0.91)	0.033
**Complications by type**				
Hemorrhagic	16 (5.8)	19 (6.9)	0.83 (0.39–1.75)	0.727
Ischemic	23 (8.4)	21 (7.6)	1.10 (0.57–2.16)	0.875
Other type	5 (1.8)	19 (6.9)	0.25 (0.07–0.71)	0.006
Procedure-related morbidity/death	23 (8.4)	25 (9.1)	0.91 (0.48–1.73)	0.880
Remedial procedure for complication	12 (4.4)	9 (3.3)	1.35 (0.51–3.69)	0.657
**Other medical complications**				
Acute hydrocephalus	29 (10.5)	16 (5.8)	1.91 (0.97–3.86)	0.061
Intracranial infection	5 (1.8)	29 (10.5)	0.16 (0.05–0.42)	<0.001
Pneumonia	50 (18.2)	82 (29.8)	0.52 (0.34–0.80)	0.002
Ulcer stress bleeding	21 (7.6)	16 (5.8)	1.34 (0.65–2.81)	0.496
DVT	13 (4.7)	16 (5.7)	0.80 (0.35–1.82)	0.703
UTI	5 (1.8)	3 (1.1)	1.68 (0.32–10.91)	0.725

EVT, endovascular treatment; MC, microsurgical clipping; DVT, deep vein thrombosis; UTI, urinary tract infection.

Regarding other medical complications, compared to those in the MC group, the EVT group had a lower likelihood of intracranial infection (OR = 0.16, 95% CI 0.05–0.42; *p <* 0.001) and pneumonia (OR = 0.52, 95% CI 0.34–0.80; *p =* 0.002), while the probabilities of acute hydrocephalus (OR = 1.91, 95% CI 0.97–3.86; *p =* 0.061), ulcer stress bleeding (OR = 1.34, 95% CI 0.65–2.81; *p =* 0.496), DVT (OR = 0.80, 95% CI 0.35–1.82; *p =* 0.703), and UTI (OR = 1.68, 95% CI 0.32–10.91; *p =* 0.725) were comparable (Table 3).

### Primary clinical outcome

**Figure 2:**
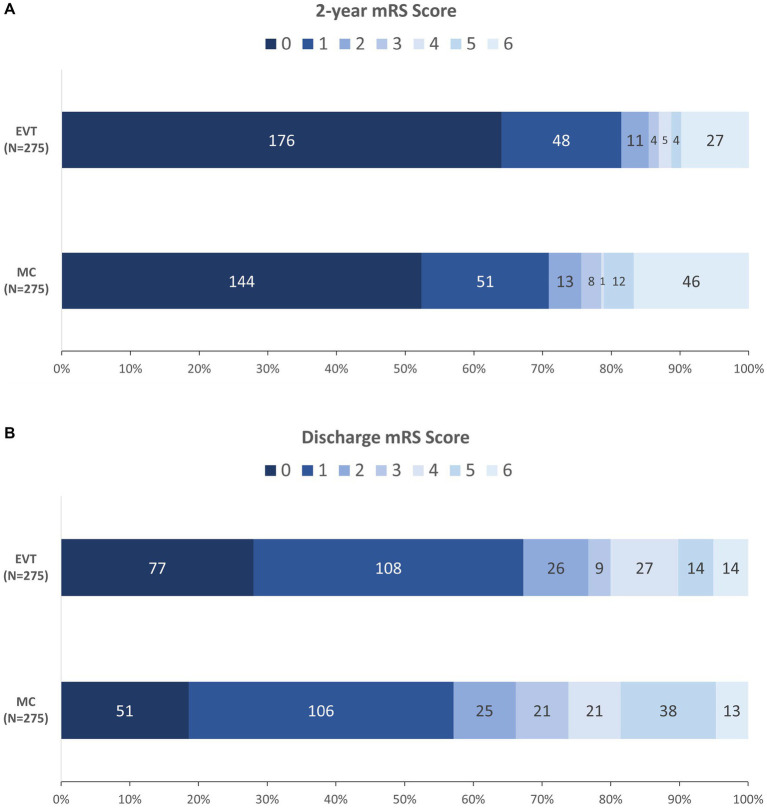
Distribution of mRS at 2 years **(A)** and at discharge **(B)** between the two groups. EVT, endovascular treatment; MC, microsurgical clipping; mRS, modified Rankin scale.

At 2 years, 235/275 (85.5%) patients in the EVT group achieved functional independence (mRS score 0–2), whereas 208/275 (75.6%) patients in the MC group achieved functional independence (OR = 1.89, 95% CI 1.20–3.00; *p =* 0.005). Figure 2A illustrates the detailed distribution of mRS scores at 2 years between the groups. Among those independent variables, the median times to recovery were 2.0 (IQR: 1.0–3.0) and 3.0 (IQR: 2.0–7.0) months (*p <* 0.001), respectively (Table 4).

**Table 4:** Primary and secondary clinical outcomes.

Outcomes	EVT (*n* = 275)	MC (*n* = 275)	OR /MD (95% CI)	*p*-value
**Primary outcome**				
Functional independence at 2 years	235 (85.5)	208 (75.6)	1.89 (1.20–3.00)	0.005
**Secondary outcomes**				
Functional independence at discharge	211 (76.7)	182 (66.2)	1.68 (1.14–2.50)	0.008
In-hospital mortality	14 (5.1)	13 (4.7)	1.08 (0.46–2.55)	1.000
2-year all-cause mortality	27 (9.8)	46 (16.7)	0.54 (0.31–0.93)	0.023
Retreatment	5 (1.8)	5 (1.8)	1.000 (0.23–4.40)	1.000
Length of stay in days, [median (IQR)]	13 (10–19)	17 (14–23)	3.9 (2.0–5.9)	<0.001
Time to independence, month, [median (IQR)]	2 (1–3)	3 (2–7)	1.4 (0.7–2.2)	<0.001

EVT, endovascular treatment; MC, microsurgical clipping; IQR, interquartile range; MD, mean difference.

### Subgroup analysis

**Figure 3:**
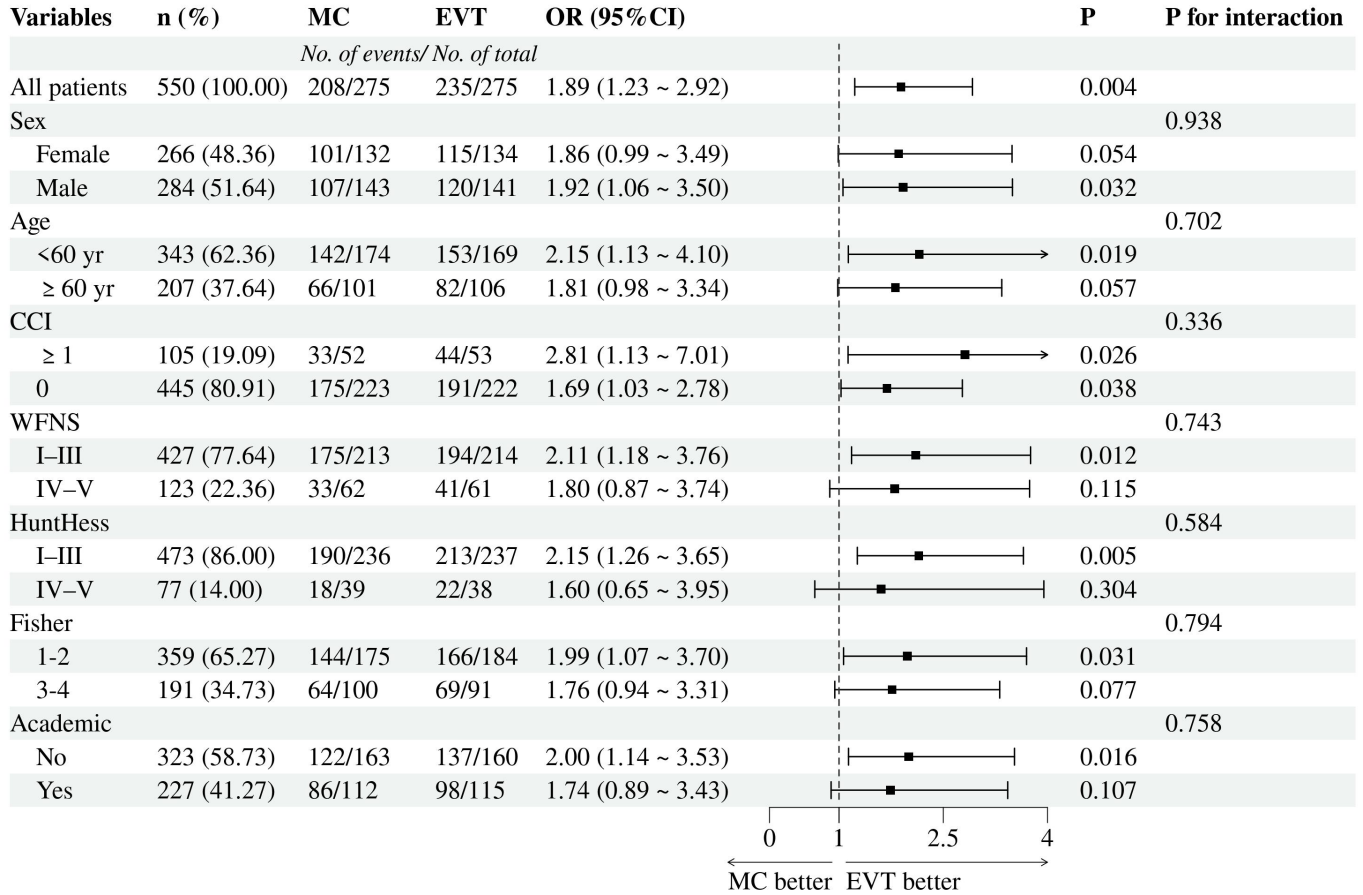
Subgroup analyses for 2-year functional independence. MC, microsurgical clipping; EVT, endovascular treatment.

The subgroup analysis of 2-year functional independence in the matched cohorts showed no evidence indicating heterogeneity of treatment effects across any prespecified subgroups, including when subgrouping by sex, age, CCI, WFNS grade, Hunt–Hess grade, Fisher grade, hospital type, or timing of treatment. The direction of effects favored EVT across all strata, although the ORs for treatment were not significant for patients with Hunt–Hess grades 4–5 or Fisher grades 3–4 (Figure 3).

### Sensitivity analysis

In the sensitivity analysis, we included the entire dataset to examine the robustness of the results obtained in the matched cohorts. At 2 years, functional independence (mRS score 0–2) was observed in 472/547 (86.3%) EVT patients and 264/346 (76.3%) MC patients (OR = 2.08, 95% CI 1.52–2.86; *p <* 0.001). Multivariate analyses revealed that EVT was associated with a higher rate of 2-year functional independence (aOR = 1.89, 95% CI 1.33–2.70; *p <* 0.001) (Supplementary Table S4).

If an unmeasured confounder were associated with the exposure and outcome with an OR of 2.09 (lower confidence limit, 1.42), it could entirely explain the association between EVT and 2-year functional independence.

### Secondary clinical outcomes

At discharge, 211/275 (76.7%) patients in the EVT group achieved functional independence (mRS score 0–2), whereas 182/275 (OR = 1.68, 95% CI 1.14–2.50; *p =* 0.008) patients in the MC group achieved functional independence. Figure 2B illustrates the detailed distribution of mRS scores at discharge between the groups. Patients in the EVT group had a lower 2-year all-cause mortality rate (OR = 0.54, 95% CI 0.31–0.93; *p =* 0.023) and a shorter length of stay (median [IQR]: 13.0 [10.0–19.0] vs. 17.0 [14.0–23.0] days, *p <* 0.001), while the in-hospital mortality (OR = 1.08, 95% CI 0.46–2.55; *p =* 1.000) and retreatment rate (OR = 1.00, 95% CI 0.23–4.40; *p =* 1.000) were similar (Table 4).

## Discussion

Although AComA aneurysms are common intracranial lesions presenting challenges for both EVT and MC, there is still limited literature comparing these two treatment modalities. Previous comparative studies have shown a significant shift in the treatment paradigm for AComA aneurysms, with the proportion of cases treated with EVT increasing from an initial 17 to 50–85% (5, 13–17). Safety and clinical outcomes have also evolved from being inferior to MC to now being comparable (5, 13–17). However, most of these studies have been single-center, small-sample, retrospective designs, with limited focus on procedural complications. As a results, previous studies have not provided sufficient evidence to establish the superiority of EVT. The optimal treatment for ruptured AComA aneurysms remains controversial. In the predominantly endovascular era, new clinical evidence is urgently needed to guide decision-making.

In this multicenter study, we utilized real-world registry data collected between 2017 and 2020 to evaluate the safety and clinical outcomes of this site-specific aneurysms treated with different treatment modalities. The results of this study show that among patients with ruptured AComA aneurysms who underwent either EVT or MC, those in the MC cohort were more prone to experiencing DC and procedural complications, while procedural morbidity/death and remedial procedure for complication were similar to those in the EVT group. Patients in the EVT group were more likely to achieve functional independence at discharge and at the 2-year follow-up (mRS score 0–2). Additionally, we observed a lower 2-year all-cause mortality rate in the EVT group than in the MC group and a comparable retreatment rate between the groups. These findings indicated that EVT for ruptured AComA aneurysms provides superior clinical outcomes and comparable safety outcomes compared to MC without increasing the rate of retreatment.

### Stent-assisted coiling

In this study, approximately 30% of patients in the EVT group were coiled with a stent-assistant, which was higher than that previously reported (less than 10%) (3, 18). Wide-necked aneurysms account for approximately 40% of AComA aneurysms (19), and there is a higher risk associated with stent implantation in patients with acute rupture (20). Although studies have shown that SAC for ruptured AComA aneurysms is feasible and safe (21–23), clinicians remain cautious and tend to prefer MC or BAC for such lesions. However, these complex morphological aneurysms pose similar challenges to neurosurgeons and may carry higher risks of procedural complications. Compared to that of BAC, the flow remodeling effect of stents enhances sustained aneurysm occlusion and simultaneously provides a climbing “scaffold” for aneurysmal neck endothelialization, thereby offering advantages in increasing occlusion rate and lowering recurrence rate and retreatment rate. In the present study, although approximately one-third of patients underwent SAC, the total rate of procedural complications in the EVT group was lower than that in the MC group (14.2% vs. 21.1%, *p =* 0.044), with comparable rates of procedure-related morbidity/death and ischemic complications (8.4% vs. 9.1%, *p =* 0.880). Importantly, the retreatment rate was not inferior to that in the MC group (1.8% vs. 1.8%, *p =* 1.000). Our findings suggest that the ongoing advancement of interventional materials and adjunctive technologies is improving the safety of EVT for treating complex aneurysms like these. However, considering intraprocedural thrombosis and postprocedural infarction occurred in 4.7 and 3.6% of the EVT patients, respectively, stent-related complications should not be underestimated. Staged stent implantation is a viable alternative for patients at greater risk for acute stent placement (24).

### Decompressive craniectomy

It has been reported that approximately 10% of patients with aneurysmal SAH undergo DC (25), with 15% of them owing to ruptured AComA aneurysms (26). However, the rate of DC for AComA aneurysms treated with different therapies has not been reported. Our study indicates that patients treated with MC were more likely to undergo DC than those treated with EVT (19.3% vs. 1.5%, *p <* 0.001). This finding is consistent with studies involving all aSAH patient populations (25, 27). Although patients with associated compressive hematomas or brain herniation are more likely to undergo clipping, there was still a significant difference in DC rates between the two treatment groups in the current study, even after strict PSM. This may reflect differences in the indications for DC between the two treatment groups, suggesting a strong correlation between DC indications and the method of aneurysm repair. For patients with intracranial hypertension and brain herniation, DC is necessary as it can be life-saving. Because it may improve the prognosis. However, for those who are admitted with a good WFNS grade and without compressive hematomas, is it possible that EVT could spare them from DC? We hope to address this question in future research.

The next step is to establish specific indications for early DC for MC patients and EVT patients, respectively. These indications may include not only indirect evidence of intracranial hypertension, such as consciousness status and radiological findings, but also such as invasive intracranial pressure monitoring and transcranial Doppler monitoring. Moreover, DC after coiling, rather than simultaneous clipping and DC, may lead to better clinical outcomes for patients with poor-grade aSAH.

### Procedural complications

In previous comparative studies involving ruptured AComA aneurysms, procedural complications were not sufficiently characterized (4, 5, 13, 14, 17, 28). In the post-BRAT study, Moon et al. (3) reported only EVT-related complications, with major complications accounting for 3.2% and minor complications accounting for 7.5%. Several meta-analyses have reported procedural complications of EVT for AComA aneurysms, but none have distinguished between unruptured and ruptured AComA aneurysms, nor have they compared EVT with MC (29, 30). Considering that each treatment modality carries its prefer set of complications, a detailed comparison of individual complication rates between the two approaches may not yield definitive conclusions. Thus, we not only enumerated various specific complications but also assessed them from different perspectives to provide a reference for future research. Finally, we found that the most common complications in the EVT group were thrombosis (4.7%), new symptom infarction (3.6%), intraprocedural ruptured (2.9%), and postprocedural intracranial hemorrhage (2.9%). In the MC group, these included new symptom infarction (8.4%), postprocedural intracranial hemorrhage (4.4%), postprocedural seizure (4.4%), and access site complication (4.4%). Although the overall rate of procedural complications in the EVT group was significantly lower than that in the MC group, there was no significant difference in the rate of procedure-related morbidity/death. The rates of hemorrhagic and ischemic complications were comparable between the EVT and MC groups in terms of the type of complications. These data from the research suggested that the safety profile of EVT for ruptured AComA aneurysms is comparable to that of MC. Our finding is in line with those of a study evaluating ruptured wide-necked aneurysms in which Mascitelli et al. (31) reported no significant differences between the EVT and MC groups in terms of procedural complications (*p* = 0.629) or neurological morbidity/death due to complications (*p* = 0.552).

The results presented here underscore several procedural complications that warrant attention. First, ischemic complications are the most common procedural complications for both EVT and MC. For EVT, thromboembolic events during coiling and new onset symptomatic cerebral infarction after coiling should be minimized. For MC, efforts should be made to protect branch arteries and avoid inducing hypotension during clipping, as Alying et al. (32) found a significant correlation between intraoperative hypotension and postoperative neurological decline. Second, postprocedural intracranial hemorrhage is also a common complication in the MC group (4.4%). Although clipping can achieve immediate aneurysm occlusion, success largely depends on anatomical features and the surgeon’s experience. Incomplete aneurysm clipping and inadequate hemostasis during surgery can lead to postoperative rebleeding, including SAH, subdural/epidural hematoma, and intracerebral hematoma. Additionally, we found that patients undergoing MC had a higher incidence of postprocedural seizure (4.4%) and higher incidence of access site complication (4.4%).

### Functional outcomes

Theoretically, over time, patients with mild to moderate disability (mRS score of 2–4) gradually recover, and those with severe disability (mRS score of 5) gradually pass away, potentially diminishing the superiority of EVT over MC in functional outcome. Moon et al. (5) found no significant difference in favorable outcomes between the two groups at a 3-year follow-up. Heit et al. (14) observed in a comparative study of 100 cases of ruptured AComA aneurysms that patients undergoing clipping were more likely to be functionally dependent at discharge (OR = 3.2; *p <* 0.05), but this difference was no longer significant at 3 months (*p =* 0.24). However, in the current study, the EVT group not only had a higher probability of functional independence at discharge (76.7% vs. 66.2%, *p* = 0.005) but also maintained a significant advantage at 2 years (85.5% vs. 75.6%, *p* = 0.008). Furthermore, the prognostic trend was consistent across all subgroups (interaction *p >* 0.05) and remained stable according to sensitivity analysis. Therefore, we conclude that EVT for ruptured AComA aneurysms offers a better functional outcome compared to MC. This finding aligns with a recent single-center analysis showing a 3-month favorable outcome rate of 73.1% in coiling group and 57.4% in clipping group (*p* = 0.016) (4). Further multivariate analysis confirmed that coiling was an independent predictor of good outcomes in patients with ruptured AComA aneurysms (OR = 3.67, 95% CI: 1.70–7.90, *p =* 0.001) (4). Similarly, the long-term outcomes of the International Subarachnoid Aneurysm Trial (ISAT) revealed that patients in the EVT group were more likely to be alive and independent at 10 years than those in the MC group (OR 1.34, 95% CI 1.07–1.67) (33).

The clear underlying reason for the discrepancy in clinical outcomes between the EVT and MC groups remains incompletely understood. Ayling et al. demonstrated that in the context of SAH, MC led to a greater perioperative decline in GCS scores, which in turn was associated with poor long-term prognosis (32). However, the limited data in this study were insufficient to support our assessment of changes in perioperative GCS scores between the groups. A large, multicenter study in Canada showed that patients with MC more commonly suffer from medical complications such as UTI, pneumonia, cardiopulmonary arrest, and seizures, all of which are associated with poor prognosis (34). Notably, we also observed more frequent incidences of pneumonia and intracranial infection; greater incidences of DC, tracheotomy and transfusion; and increased hospital stays in the MC group. These results collectively imply that MC patients may have experienced worsening of their general condition after clipping and may have required more time to recover, indicating a more profound physiological impact.

In the subgroup analysis of the primary outcome (Figure 3), all characteristics did not modify the treatment effect (P for interaction >0.05), including patients aged <60 years or those with poor-grade (WFNS IV-V or Hunt-Hess IV-V). Some clinicians are concerned about the treatment of EVT for young patients because of the corresponding higher rates of recurrence and retreatment associated with it. However, our data indicated that, compared to concerns about long-term recurrence, prioritizing short-term favorable outcomes may be more beneficial. Moreover, despite the higher recurrence rate associated with EVT for AComA aneurysms, the majority do not necessitate retreatment (35). Catapano et al. (18) found that AComA aneurysms >7 mm were significantly more likely to recur (22.2% vs. 6.7%, *p* = 0.005), while aneurysms smaller than 4 mm did not require retreatment. Therefore, we believe that the emphasis on recurrence of AComA aneurysms after coiling should be balanced, and regular postoperative angiographic follow-up is crucial. Besides, the superior prognosis of EVT over MC was more pronounced in non-academic hospitals. This finding might reflect differences in the proficiency of surgeons across different hospital types. MC is known to have a steeper learning curve, and is relatively more challenging to disseminate. Neurosurgeons in academic hospitals may be more adept at clipping than their counterparts in non-academic hospitals. Therefore, we suggest that surgeons consider their own proficiency when selecting the optimal therapy modality. After all, experience is the best teacher.

### Strengths and limitations

The main strengths of this study lie in its multicenter design and the use of propensity score matching, which greatly enhance the generalizability of our findings. However, this study has several limitations. First, due to the retrospective, observational design of this study, patients were not randomized to MC or EVT, and treatment decisions were influenced by individual and institutional biases. While we used PSM to address selection bias, the results may still be affected by unmeasured confounders. To further examine potential bias related to selection and unmeasured confounders, we conducted a sensitivity analysis. The E-value for the observed OR and confidence limit were 2.09 and 1.42, respectively. Although the E-value we obtained suggests a robust association, we are unaware of any potential confounders of this magnitude that are not already accounted for in the model. Second, the population of this study were limited to China, where most patients were discharged home instead of being sent to community rehabilitation facilities for further rehabilitation. This practice might prolong the recovery period for patients who are functionally dependent at discharge or even hinder their reintegration into society. Therefore, it is advisable to conduct similar studies in diverse populations to evaluate the generalizability of the findings across different settings. Third, 181/1074 (16.9%) of patients were lost to follow-up in this study. However, for the patients lost to follow-up, the likelihood of a favorable outcome at discharge was significantly greater in the EVT group than in the MC group (Supplementary Table S2). Thus, we believe that the better outcomes found in the EVT group are unlikely to be explained by loss to follow-up. Finally, we did not evaluate the patients by angiographic follow-up, which is another aspect of therapeutic efficacy, but retreatment rate was reported.

## Conclusion

In summary, as the largest comparative study for ruptured AComA aneurysms to date, this study provided a realistic depiction of treatment experiences for this lesion in the real world. Our results revealed that EVT for ruptured AComA aneurysms provides superior clinical outcomes and comparable safety compared to those of MC without increasing the rate of retreatment. These findings may help guide personalized treatment decisions for patients with ruptured AComA aneurysms. In particular, prioritizing EVT may be more beneficial when both EVT and MC are viable treatment options. However, given the limitations, additional studies in other countries are needed to verify this finding.

## Data availability statement

The original contributions presented in the study are included in the article/Supplementary material, further inquiries can be directed to the corresponding authors.

## Ethics statement

The studies involving humans were approved by the Ethics Committee of Tianjin Medical University General Hospital. The studies were conducted in accordance with the local legislation and institutional requirements. The ethics committee/institutional review board waived the requirement of written informed consent for participation from the participants or the participants’ legal guardians/next of kin due to the observational/retrospective nature of the study.

## Author contributions

MY: Conceptualization, Data curation, Formal analysis, Investigation, Methodology, Writing – original draft. YL: Data curation, Formal analysis, Investigation, Methodology, Project administration, Software, Writing – original draft. JL: Methodology, Validation, Writing – review & editing. XA: Data curation, Investigation, Software, Validation, Writing – original draft. HL: Data curation, Investigation, Validation, Writing – review & editing. BW: Data curation, Formal analysis, Project administration, Supervision, Writing – review & editing. YZ: Methodology, Validation, Writing – review & editing. XZ: Data curation, Writing – original draft. CH: Data curation, Writing – original draft. LH: Data curation, Resources, Writing – review & editing. XY: Funding acquisition, Project administration, Resources, Supervision, Writing – review & editing. JY: Data curation, Resources, Supervision, Writing – review & editing.
